# Emerging Nano-/Biotechnology Drives Oncolytic Virus-Activated and Combined Cancer Immunotherapy

**DOI:** 10.34133/research.0108

**Published:** 2023-04-03

**Authors:** Chao Fang, Gaozhe Xiao, Taixia Wang, Li Song, Bo Peng, Bin Xu, Kun Zhang

**Affiliations:** ^1^ Central Laboratory and Department of Urology, Shanghai Tenth People’s Hospital, Tongji University School of Medicine, Tongji University, No. 301 Yan-chang-zhong Road, Shanghai 200072, China.; ^2^ National Center for International Research of Bio-targeting Theranostics, Guangxi Medical University, No. 22 Shuangyong Road 22, Nanning, Guangxi 530021, China.; ^3^ Department of Urology, Shanghai Ninth People’s Hospital, Shanghai Jiaotong University School of Medicine, No. 639 Zhizaoju Road, Huangpu, Shanghai 200011, China.

## Abstract

Oncolytic viruses (OVs) as one promising antitumor methods have made important contributions to tumor immunotherapy, which arouse increasing attention. They provide the dual mechanisms including direct killing effect toward tumor cells and immune activation for elevating antitumor responses, which have been proved in many preclinical studies. Especially, natural or genetically modified viruses as clinical immune preparations have emerged as a new promising approach objective to oncology treatment. The approval of talimogene laherparepvec (T-VEC) by the U.S. Food and Drug Administration (FDA) for the therapy of advanced melanoma could be considered as a milestone achievement in the clinical translation of OV. In this review, we first discussed the antitumor mechanisms of OVs with an emphasis on targeting, replication, and propagation. We further outlined the state of the art of current OVs in tumor and underlined the activated biological effects especially including immunity. More significantly, the enhanced immune responses based on OVs were systematically discussed from different perspectives such as combination with immunotherapy, genetic engineering of OVs, integration with nanobiotechnology or nanoparticles, and antiviral response counteraction, where their principles were shed light on. The development of OVs in the clinics was also highlighted to analyze the actuality and concerns of different OV applications in clinical trials. At last, the future perspectives and challenges of OVs as an already widely accepted treatment means were discussed. This review will provide a systematic review and deep insight into OV development and also offer new opportunities and guidance pathways to drive the further clinical translation.

## Introduction

Oncolytic viruses (OVs) can be considered as oncolytic agents, which are usually divided into 2 main categories, i.e., naturally occurring and genetically modified OVs. Genetically modified viruses are obtained using genetic engineering technology to engineer common viruses and disable their ability to infect normal cells, which allows the specific lysis of tumor cells. OVs retain the high invasiveness, robust replication, and rapid propagation abilities of the original virus in tumors, while in normal cells, viral replication and expansion are hampered since tumor cells have an abnormal metabolism and a differential microenvironment compared to normal cells [1]. OVs can target and kill tumor cells after expansion, consequently achieving antitumor effects. Furthermore, OVs potentiate tumor cell death by promoting immune cell activation and recruitment to target any residual tumor cells. These appealing features dictate the prevalence of OVs in different scientific communities, akin to nanomedicine that has developed several nanotechnologies to counteract tumor progression [2–11].

The earliest OVs can be traced back to a century ago. It has been gradually recognized that although OVs can kill cancer cells, the comprehensive action mechanism has not been completely elucidated due to the complicated biological activities of OVs yet, e.g., their involvement roles in innate and adaptive immune responses, antiviral responses, thrombi, and mitigation of the immunosuppressive microenvironment [12–14]. Over the past 2 decades, the favorable efficacy of lysis viruses has been demonstrated to benefit cancer patients in extensive clinical trials [15–17]. Researchers have proposed various viruses as vectors and engineered them into OVs for immunotherapy [18]. Typically, adenovirus, poxvirus, herpes simplex virus type 1 (HSV-1), coxsackie virus, measles virus, Newcastle disease virus (NDV), and eutherian virus have been evaluated in early clinical trials. Encouragingly, several OV drugs have received regulatory approvals for entry into clinical trials, e.g., Rigvir, talimogene laherparepvec (T-VEC), and Oncorine (i.e., H101). In particular, T-VEC is a herpes-based oncolytic vector approved by the U.S. Food and Drug Administration (FDA) for the treatment of metastatic melanoma, and H101 is a genetically modified oncolytic adenovirus that is used in combination with chemotherapeutic drugs to treat nasopharyngeal carcinoma [19].

OVs can be divided into 2 types according to their variable genetic materials, i.e., single- or double-stranded DNA and RNA, and both types of viruses can infect host cells and undergo adaptive replication in vivo (Table 1). According to the different genes encoded by different viruses, pathogenic properties, and host immune system responses, the results of viral infections are variable [20]. Based on the different properties of various viruses, appropriate OV vectors with certain characteristics have been selected to treat specific types of cancer. In this regard, OV-based tumor regression does not require the inclusion of specific antigens in the vector, which is different from the prerequisites demanded by common virus-based “vaccines.” Unfortunately, viral replication and persistent infection of tumor cells are neutralized by inherent antiviral responses [21–23]. The tug-of-war between the 2 opposing forces and the intricate interplay between them could affect the efficacy of tumor treatment. At the same time, more and more OVs are entering the clinical development stage, and their drug delivery efficiency will also be a key factor affecting the optimal therapeutic effect. With the rapid development of nanomaterials, nanocarriers can establish new active delivery modes and greatly improve the delivery efficiency of OVs to improve the delivery barriers of traditional viruses [24–26]. In this review, we highlighted key concerns in the development of OV-based immunotherapy. Adequate evidences that support OVs as immunotherapeutic agents for cancer therapy in preclinical or clinical trials were presented. Additionally, we discussed some of the unique challenges related to development of new OVs.

**Table 1.** Properties of key oncolytic viruses.

	**Newcastle disease virus**	**Herpes simplex virus-1**	**Vaccinia virus**	**Adenovirus**	**Coxsackie viruses**	**Maraba virus**	**Reovirus**
Particle size	100–500 nm	~200 nm	70–100 nm	70–90 nm	~28 nm	80–95 nm	60–80 nm
Genome	ss (−) RNA	dsDNA	dsDNA	dsDNA	ssRNA	ss (−) RNA	dsRNA
Genome size	~15 kb	152 kb	190 kb	25–45 kb	~8 kb	11–15 kb	18–23 kb
Transgene capacity	+	++	++	+/−	−	−	N/A
Pathogenicity of native virus	Hemorrhagic damage to the digestive tract, transitory conjunctivitis	Gingivostomatitis, keratoconjunctivitis, encephalitis	Severe pneumonia, skin impetigo	Fever, eye infection, acute respiratory diseases, gastroenteritis	Aseptic meningitis, HFM disease, herpetic angina, myocarditis	Flu-like illness	Respiratory tract infection, gastroenteritis, diarrhea
Viral immunogenicity	−	−	−	−	−	−	−
Blood–brain barrier penetration	−	−	+	−	+/−	−	+
Virulence of wild-type virus	+	−	+/−	+/−	+/−	+	+
Achievable titer (PFU per ml)	10^8^	10^10^	10^9^	10^12^	10^9^	2 × 10^10^	10^9^

## Oncolysis Mechanism of OVs

Ideal OVs should satisfy several demands: (a) high targeting or affinity toward tumor cells, (b) rapid replication and expansion, (c) attenuated antiviral responses, and (d) activated immune responses. Demands (a) to (c) have been reviewed and summarized in many reports, while demand (d) receive few reviews, and especially those cutting-edge progress in OVs in combination with other therapeutic methods or technology has been rarely reviewed, e.g., nanotechnology and immune checkpoint blockade (ICB) immunotherapy. Although the scope of this review is not to focus on features (a) to (c) of OVs, the basic knowledge of OV oncolytic mechanisms of actions needs to be explained to allow the readers to fully understand how OVs boost the activation of an immune response against cancer cells and the effects on combining OVs with several immunotherapies.

### OV targeting strategies

The potential of tumor-targeted therapy has elicited increasing interest relating to tumor treatment in both clinical and scientific research. On this account, improving the targeting and proliferative efficiency of OVs is the primary concern of researchers as they focus on designing OVs to execute antitumor actions. Although some natural viruses are equipped with tumor-targeting ability, most viruses need genetic engineering to express certain proteins to bind with the specific receptor overexpressed on tumor cells, which imparts OVs with high affinity to tumor cells. Tumor cells are genetically and physiologically characterized by the gain or loss of some functions or the up- or down-regulations of some gene functions, which distinguishes these cells from normal cells in the body. Additionally, tumor-specific promoters can drive the expression of genes necessary for viral replication and confine viral replication to tumor cells. Extensive evidences are now available to better improve the OV targeting efficacy aiming at tumors.

**Fig. 1.**
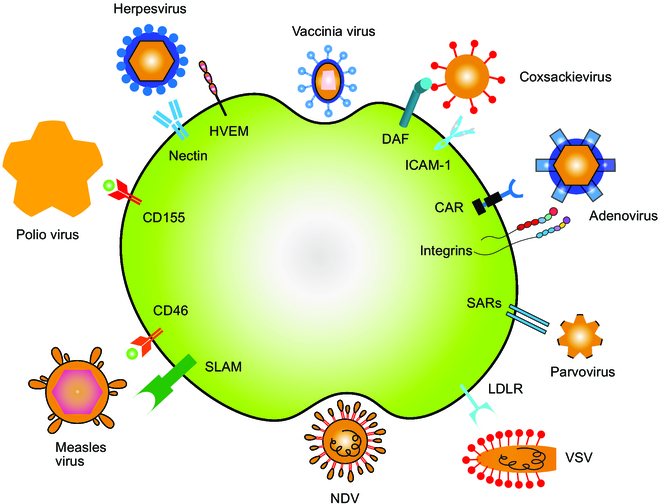
OVs enter tumor cells through different approaches. Cellular receptors overexpressed on the surface of cancer cells are the main targets exploited by OVs for entry into tumor cells. Some viruses can target multiple receptors, while some receptors can act as targets for multiple viruses. Endocytosis through membrane fusion and syncytium formation is also a pathway by which some viruses enter cells.

The abnormally expressed proteins on the surface of tumor cells are responsible for regulating the tumor tropism of many OVs in current clinical situations, as shown in Fig. 1 [27]. As a paradigm, herpesvirus entry mediator (HVEM) and nectin-1 are the targets of HSV-1 entry into cells, and these surface receptors are overexpressed on some cancer cells (e.g., melanoma cells) [28]. Measles does preferentially infect tumor cells since CD46, as another surface receptor, is up-regulated in tumors and a certain threshold of expression is required for entry [29]. Thereby, CD46 has been reported as the target molecule for mediating measles virus entry into cancer cells [30], which could prevent cell clearance by inactivating the complement pathway of immune system. Moreover, diverse retargeted measles viruses have been developed in preclinical and clinical trials [31,32]. Overexpression of intercellular adhesion molecule 1 (ICAM-1; also known as CD54) and decay acceleration factor (DAF; also known as CD55) in melanoma, multiple myeloma, and breast cancers allows entry of coxsackie viruses into tumor cells [33–35].

It is noted that live attenuated measles viruses are not tumor specific since the natural receptors for these viruses, i.e., CD46 [36] and signaling lymphocyte-activation molecule (SLAM) [37–39], are ubiquitously distributed. To overcome this limitation, a strategy is used to detarget the OVs from their native receptor and engineer them to express ligands such as single-chain antibodies that can recognize receptors specifically overexpressed on cancer cells. Nakamura and coworkers [40] exploited a pseudo-receptor system in which viruses could be retargeted to tumor-selective CD38, epidermal growth factor receptor (EGFR), or mutant EGFR VIII (EGFR VIII) after disabling the standard targeting receptors to efficiently enter CD46- and SLAM-positive cells in vivo. This system was available for generating retargeted measles viruses with the maximally optimized therapeutic efficacy of nontargeted OVs, indicating that this method could be preferably extended to improve the dissemination of retargeted viruses in other virus families. Certainly, the retargeting strategy is not unique, such as HSV, and several examples of HSV-1 retargeting to breast and glioblastoma multiforme have been documented [41–43]. Reliable in vivo data have been provided to validate the tumor retargeting of these OVs and their destructive actions against tumor tissues via the tumor lysis pathway [39].

### OV attenuation

**Fig. 2.**
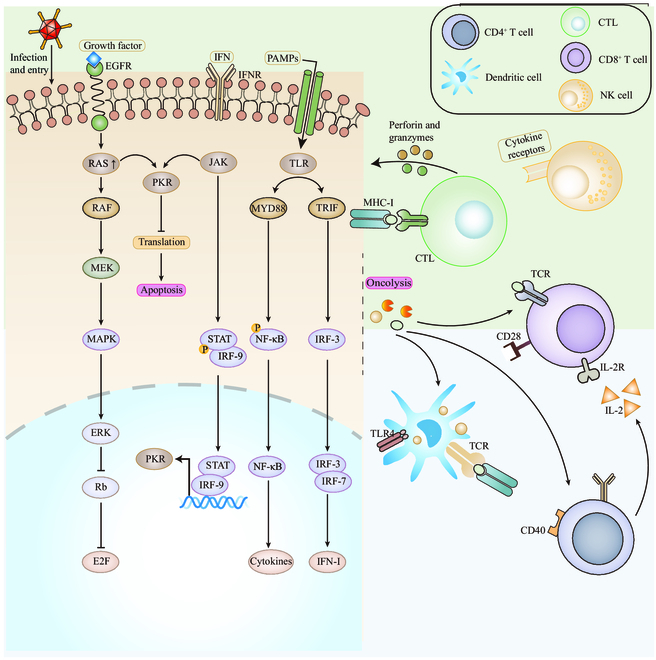
Viruses induce adaptive immunity. OVs stimulate immunity through tumor cell lysis and presentation of viral or tumor antigens to exert oncolytic effects. OVs also evoke an immune response, triggering more immune cells to further lyse any remaining tumor cells.

The self-replication ability of OVs is the natural feature of viruses, and the main reasons why OVs can preferably infect tumor cells and replicate in tumor cells is the dysfunction and aberrant signaling pathway of tumor cells. In detail, the aberrant signaling pathway make tumor cells fail to perceive and block virus replication. Cancer-specific aberrations in RAS, TP53, RB1, and PTEN genes and other cancer-related genes that encoded WNT signaling-mattering factors and predisposed cancer cells to viral infection have been identified. After viral infection of tumor cells, cell cycle regulatory proteins, oncogenes, tumor suppressor genes, and key signaling components in innate signaling pathways in tumor cells including IRF3 and STAT are activated [44,45]. They limit the detection of viral particles by Toll-like receptors (TLRs) and Janus kinases (JAKs), making it easier for the virus to replicate and undergo oncolysis. Viruses will lead to down-regulation of interferon-1 (IFN-1) and pro-inflammatory cytokine production. OVs subsequently cause oncolysis, which release pathogen-associated molecular patterns (PAMPs) and tumor-associated antigens (TAAs), among others. PAMPs stimulate the immune system by activating the related receptors such as TLRs. In an immunostimulatory environment, TAAs are taken up and released by antigen-presenting cells (such as dendritic cells) to generate specific cytotoxic CD8^+^ T cells and induce local and systemic antitumor immunity (Fig. 2). Among OVs, modified strains of the rhabdoviruses vesicular stomatitis virus (VSV) and Maraba virus are the representatives of viruses that rely on this pathway [12,46]. More significantly, the deficit of IFN in tumor cells fail to arouse the antiviral defense system and make tumor cells sensitive to viruses, while in normal cells, IFN and IFN-related factors may inhibit virus replication and clear away viruses. Also, the large amounts of nucleotide as the arsenal in tumor cells can supply sufficient substance that virus replication demands, and the tumor cell apoptosis inhibition by aberrant signaling pathway (e.g., PKR) provides sufficient time for the replication and oncolysis action of OVs. Generally, in genetically engineered OVs, genes favorable for replication in normal cells are deleted, while genes mattering replication in tumor cells are preserved, and this genetic engineering approach is considered to be a targeting strategy for OVs [47] In addition, tumor-driven mutations specifically increase the selectivity of viral replication in tumor cells.

### OV arming strategies

To ensure biosafety, appropriately attenuating host antiviral immune response against OVs has been considered as a strategy to facilitate OV’s oncolytic activity. However, antiviral immune attenuation also inevitably impairs the innate immunity, determining that antiviral immune attenuation is a double-edge sward. Here, IFN regulation is identified as an effective pathway. Xiao and coworkers [48] found that DNA-dependent protein kinase (DNA-PK) inhibitor could block IFN secretion and antiviral response, which made tumor cells sensitive to OVs for promoting antitumor oncolysis effect. In recombinant rhabdoviruses, IFN antagonist was expressed by OVs to bind with IFN-binding decoy receptor, circumvent antiviral immune responses, and promote virus replication [49]. Additionally, cell–cell adherence enhancement is also encouraged to attenuate antiviral immunity. As a paradigm, Xu and coworkers [50] engineered an oncolytic HSV to express E-cadherin (CDH1), which encoded E-cadherin. CDH1 overexpression could enhance the adherence between cells, elevate virus propagation, and simultaneously bind with the receptors overexpressed on natural killer (NK) cells to evade the antiviral activity of NK cells, which eventually promoted intercellular viral infection and accelerated viral entry for enhancing oncolysis efficiency.

By taking into consideration the above discussions, the inherent inadequacy of natural viruses, such as poor specific targeting and replication in tumor cells, activated antiviral innate immunity, and poor systematic immune responses, encourages more recombinant OV development to improve its antitumor effect. Here, genetic engineering strategy was used to insert or knock down various genes in OV vectors, imparting the recombinant OVs with various merits. Typically, the knockdown of genes in E1B-5SkD and E3 of H101 disabled H101 to infect and replicate in p53 normal cells. However, inserting certain gene is currently dominant in genetically engineered OVs. In summary, recombinant OVs can be divided into several types according to their different purposes, i.e., enhanced affinity or targeting toward tumor cells, enhanced replication and expansion, enhanced oncolysis effect, attenuated antiviral immune responses, and enhanced immune responses. Typically, E-cadherin protein-expressed recombinant OVs can attenuate antiviral immunity [50], and fibroblast growth factor 2 (FGF2)-expressed OVs was validated to potentiate the sensitivity of tumor cells toward OVs and promote oncolysis effect [51].

Generally, preferably infecting tumor cells and enhancing immune responses are 2 prevalent directions of recombination OV development. Retargeting OVs to enter cells via tumor-specific receptors is primarily highlighted, wherein the insertion of corresponding genes into the OVs enables the expressions of certain ligands capable of preferably binding with the specific receptors (e.g., CD38, EGFR, and RGD) overexpressed on tumor cells [40,52]. In terms of enhancing immune responses, various engineered OVs have been designed and developed to express certain immune stimulus factors or proteins, immune checkpoint molecules, adjuvants, or cytokines, e.g., granulocyte-macrophage colony stimulating factor (GM-CSF) and Gal [53], programmed cell death protein-1 (PD-L1) and GM-CSF [54], CD19 [55], interleukin-2 (IL-2) and IL-12 [56], and PD-L1 [57]. These overexpressed substances not only enhance immune responses but also promote the combined immunotherapy. Intriguingly, the secreted substance by recombinant OVs sometimes exerts dual functions, e.g., IFN antagonist-expressed OVs after gene engineering not only attenuated antiviral immune response but also promoted replication and expansion of OVs [49]. The recombinant vaccinia OVs that secreted CD19 protein contributed to the enhanced replication and expansion on the one hand and simultaneously bind with CD19 CAR-T cells for promoting CAR-T immunotherapy on the other hand [55].

In the course of genetic engineering of OVs, the CRISPR–Cas9 system serves as a convenient, flexible, and precise genome editing tool [58,59]. Cas9 usually refers to *Streptococcus pyogenes* Cas9 (SpCas9) [60], which harnesses a guide RNA to recognize the upstream or downstream regions of the protospacer adjacent motif (PAM) in the target sequence and allow double-strand breaks in the objective DNA sequence [61,62]. This genome editing system provides a powerful platform for engineering recombinant OVs [63]. Nowadays, several functions including enhanced targeting and oncolysis capabilities have been realized on recombinant OVs engineered with the CRISPR-Cas9 system [64]. Ni and coworkers [65] engineered 2 ICP6-mutated oncolytic HSVs (oHSVs) that were conferred with preferential killing ability against lung tumor cells, displaying notable antitumor activity and attenuated virulence. Additionally, CD40L was engineered into oHSV by CRISPR-Cas9-based gene editing, which was validated to regulate tumor microenvironment (TME) and promote dendritic cell (DC) maturation and cytotoxic T cell activation, which was available for enhancing immune responses and promoting ICB immunotherapy [66].

Oncolytic therapy using genetically engineered OVs is the way forward, where immune inhibitors or phagocytes can be united to circumvent the host immunity, facilitate OV propagation in tumors, enhance the oncolysis efficacy of OVs, and activate the systematic immunity. With accumulative advances in understanding OV-based antitumor mechanisms, activated antitumor immune responses, and gene editing technology and effective target discovery, OV therapy and combination therapy with immunotherapy or other treatment methods will evolve into promising and general approaches to achieve robust antitumor activity in the clinics.

## OV-Activated Immune Responses for Realizing Immunotherapy

### Mechanism of OV-activated immunotherapy

**Fig. 3.**
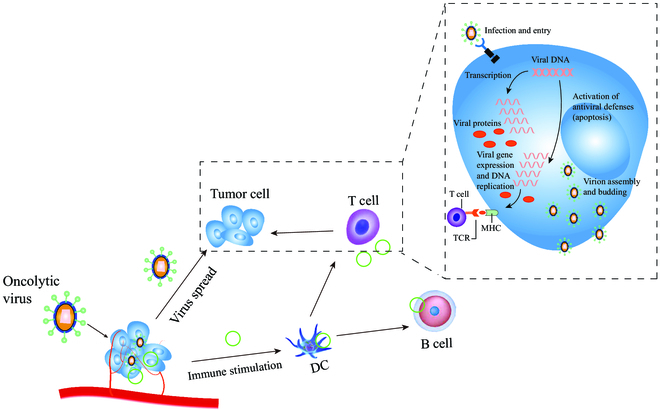
Schematic diagram of OV entering tumor tissue and exerting its effect. OVs can replicate massively inside tumors to lyse tumor cells. At the same time, it can stimulate the initiation and regulation of the body’s immune system.

**Fig. 4.**
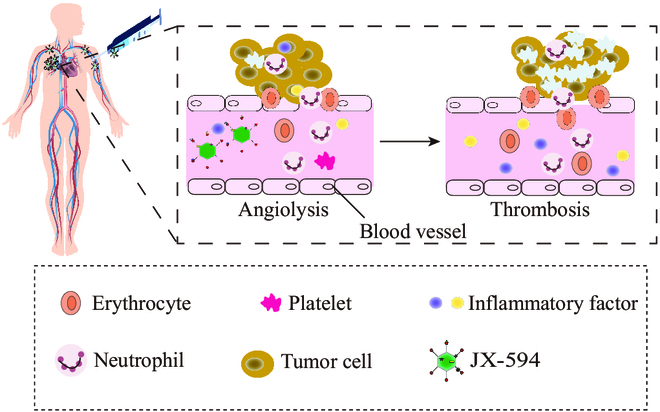
OV selectively infects and replicates itself in tumor cells, destroying tumor cells and driving the release of pathogen-associated molecules (PAMPs), tumor-associated antigens (TAAs), inflammatory factors, chemokines, and other substances, which can directly or indirectly kill tumor cells. In tumor vessels, neutrophils can enrich to form inflammatory thrombi, destroying new tumor vessels.

In addition to mediate direct oncolysis, OVs can exert anticancer effects by stimulating antitumor immune responses [67]. Therefore, OVs are also identified as a new type of tumor immunotherapy drug. The immune effects of OVs are mainly expressed through the following impacts: (a) OVs directly infect and lyse tumor cells without destroying normal cells. Viruses can preferably replicate in tumor cell lines and cause tumor cell lysis, which directly kills tumor cells and creates space for the proliferation and infiltration of immune cells (Fig. 3). (b) OVs cause the release of tumor antigens to stimulate the body’s immune response, where OVs as an in situ vaccine can kill tumor cells, causing the release of intracellular TAAs, PAMPs, and damage-associated molecular patterns (DAMPs). Moreover, OVs can infect antigen-presenting cells (APCs), promote their functional maturations, and induce the type 1 IFN responses [68]. For example, NDV-induced necroptosis in glioma cells (sensitive to necrostatin-based RIPK1 inhibition) has been found to cause the surface exposure of “eat me” signal calreticulin and the release of high mobility group box 1 (HMGB1) and generate a close correlation with potent anti-glioblastoma immunity driven by antigen-specific CD8^+^ T cells [69]. (c) OVs regulate the TME to circumvent immunosuppression. Typical suppressive cells include tumor-associated macrophages (TAMs) and myeloid-derived suppressor cells (MDSCs), both of which secrete potent immunosuppressive factors, such as IL-10, transforming growth factor-β (TGF-β), indoleamine 2,3-dioxygenase (IDO), and arginase. These factors inhibit the most important immune processes, including DC maturation, antigen presentation, inflammatory factor synthesis, and cytolytic factor function. To address them, OVs can neutralize immunosuppression and significantly alter the TME by inducing the potent proinflammatory T helper 1 (T_H_1) cell-polarizing immune responses [70]. (d) Genetically modified viruses can stimulate T cells to produce chemokines. Promoting tumor cell recognition by DCs can up-regulate the expression of chemokines, such as chemokine (C-X-C motif) ligand 1 protein (CXCL1) and chemokine (C-X-C motif) ligand 5 protein (CXCL5), and increase the infiltration and proliferation of immune cells at the site of tumor [71]. Komorowski and colleagues [71] delivered a CXCR4 antagonist expressed in the murine Fc fragment of immunoglobulin G2a (IgG2a) via OVV (OVV-CXCR4-A-Fc). In this study, it was shown that DC vaccines loaded in Dox-treated NXS2 tumor cells efficiently induced antitumor immune responses in vaccinated mice on both prophylactic and therapeutic settings [72,73]. Furthermore, they developed a therapeutic vaccine, and its antitumor efficacy was largely dependent on the reduction of tumor burden and TME reprogramming by OVV-CXCR4-A-Fc, suggesting that armed OVV could enhance vaccine-induced immune responses. (5) Endothelial cells in the tumor vasculature are sensitive to OVs (e.g., JX-594), and OVs can infect APCs to inhibit tumor angiogenesis and induce inflammatory thrombosis [74], as shown in Fig. 4. Studies have shown that VSV can directly infect and destroy tumor blood vessels in vivo without affecting normal blood vessels. VSV spreads and replicates in tumor tissue, initiates the neutrophil-mediated inflammatory responses in the body, and leads to microthrombi formation in tumor blood vessels. Microthrombi in tumor blood vessels can affect the blood vessels and block nutrient supply to tumor tissues, eventually leading to tumor cell death due to starvation [75,76].

Although immuno-oncolytic therapy mediated by the intravenous injection of OVs holds great promise, there are still some bottlenecks, i.e., restricted intratumoral replication and inherently immunotolerant microenvironment. Viral replication is restricted in 2 ways: one is the natural antiviral immunity that restricts the ability of viruses to infect tumor cells and replicate in tumor cells, and another is the early apoptosis of virus-infected cells that appose to viral replication [77]. To address the 2 unfavorable issues, immunosuppressants such as cyclophosphamide (CPA) were used to suppress antiviral immunity in some specific trials [78]. Also, cell vehicles available for loading OVs were also preferable to avoid OV clearance by the body’s immune system in some specific trials [79]. As a paradigm, researchers used endothelial progenitor cells loading the oncolytic measles virus to evade clearance of antiviral immune serum and achieve replication in tumors [80,81]. At present, many viruses have been developed with different engineering strategies to avoid immunologic surveillance by innate and adaptive immune systems, providing approaches to allow OVs to enduringly avoid immune-mediated clearance, which thus leave adequate time for the OV to generate potent antitumor immune responses.

### Antiviral response counteraction for boosting OV-activated immunotherapy

OVs exert their therapeutic effects by preferentially lysing tumor cells and inducing violent immune responses in the body, which is a promising means for tumor immunotherapy. Although antiviral response attenuation is detrimental for innate immune activation, it favors OV replication in cancer cell and oncolysis-activated adaptable immunity. To counteract the body’s antiviral responses and enhance viral replication, researchers have introduced and studied many pathways such as antibody neutralization [23], complement system [82], IFN inhibition [51], and NK cell activation [83].

The activation of immunotherapy is premised on the replication characteristics of OVs. Nowadays, no matter whether patients receive intravenous or intratumoral injection of one therapeutic OV in clinical practices [53,84], the number of viral particles that ultimately reach the cancer cells exceeds that in normal cells (i.e., IFN-α/type I IFN-positive cells) [85], and this differs significantly for different treatment settings and individual patients. OV replication in normal tissues is usually attenuated or even inhibited due to the inability of OVs to antagonize the normal cellular IFN-α/type I IFN-mediated antiviral responses [49,86]. The absence of IFN-α/type I IFN responses is found in many tumor cells during malignant evolution, which allows these tumor cells to become the ideal hosts for OV replication [87–89]. Selective invasion of both normal and cancer cells by OVs provides a distinctive advantage in activating the immune system. Therefore, developing an OV that initiates infection with a relatively small number of viral particles but has a strong ability to spread and expand within cancer cells is concluded to be a successful therapeutic approach and deserves explorations.

Immunotherapy outcome is significantly decided by the replication ability of OVs. The replication and propagation of OVs in tumor can be selectively boosted using some small molecules or gene drugs [49,90–92]. Here, by screening the small-molecule library, Xiao and coworkers [48,93] identified the M1-mediated OV sensitizers, i.e., DNA-PK inhibitors. Relying on the DNA-PK activation-dependent intrinsic immune response to IFN regulatory factor 3 (IRF-3) [94,95], the combined DNA-PK inhibitors blocked DNA-PK pathway and decreased IRF-3 to promote the proliferation and expansion of OV M1 in tumor cells. In a recent study, the Weinberger group [96] proposed a strategy to disrupt the feedback loop of herpesviruses using an engineered DNA duplex, causing the host cell to “overload” and die and also dismantling the barrier to drug resistance.

Natural body antiviral activity can hinder the therapeutic effect of OVs. Distant tumor metastasis is a dismal prognosis, and although OVs can be delivered to tumor by intravenous injection, their efficacy is diminished when transmitting through complement-depleted plasma [53]. This undesired antiviral activity involves interactions with complement factors in a calcium-dependent manner and could be facilitated by antibody neutralization with preimmune IgM. To tackle it, based on the inactivations of complement C3 component by HSV glycoprotein C and the certain humoral antiviral functions by nonimmune and immune Fc IgG bindings [97,98], Ikeda and coworkers [22] loaded the “prodrug” CPA in recombinant HSV-1 vectors to realize anticancer and mitigate immunosuppressive effect. This CPA-loaded HSV-1 and other HSV mutants harvested safer and more potent antitumor performance than hrR3 alone, and CPA continuously inhibited antibody neutralization over 4 d. Further trial was carried out by Chase and coworkers [99], and they developed recombinant HSV-1 by knocking out Hsrr and inserting CYP2Bl transgene, where the mutant HSV-1 selectively replicated in tumor cells and significantly activated the CPA prodrug to boost CPA antitumor efficiency on subcutaneous tumor xenografts.

The biosafety of biomolecules such as small interfering RNA could be significantly enhanced if OVs were able to selectively replicate into higher load in tumor tissues than in normal tissues after intravenous administration [100]. After activating the EGFR/Ras pathway in tumor cells, JX-594 as an oncolytic poxvirus can enter the tumor cells and proceed to undergo replication, transgene expression, and amplification, eventually inducing cancer cell lysis and triggering antitumor immunity elevation. Breitbach and coworkers [53] injected JX-594 intravenously into patients at different doses. They divided 23 patients into 6 groups by intravenously administering the drug with varied doses ranging from 10^5^ plaque-forming units (PFU) kg^−1^ to 10^7^ PFU kg^−1^. The most common treatment-related adverse events were grade 1 to 2 influenza-like symptoms, e.g., fever lasted 24 h. The replication ability of poxvirus is dependent on the EGFR/Ras signaling pathway, which is generally activated in epithelial cancers [1,101]. Although this trial did not find evidently improved therapeutic efficacy using this modified poxvirus, systemic delivery of the poxvirus OV by this method favored high tumor targeting and specific replication.

### Recombinant OV-enhanced immunity effect

**Fig. 5.**
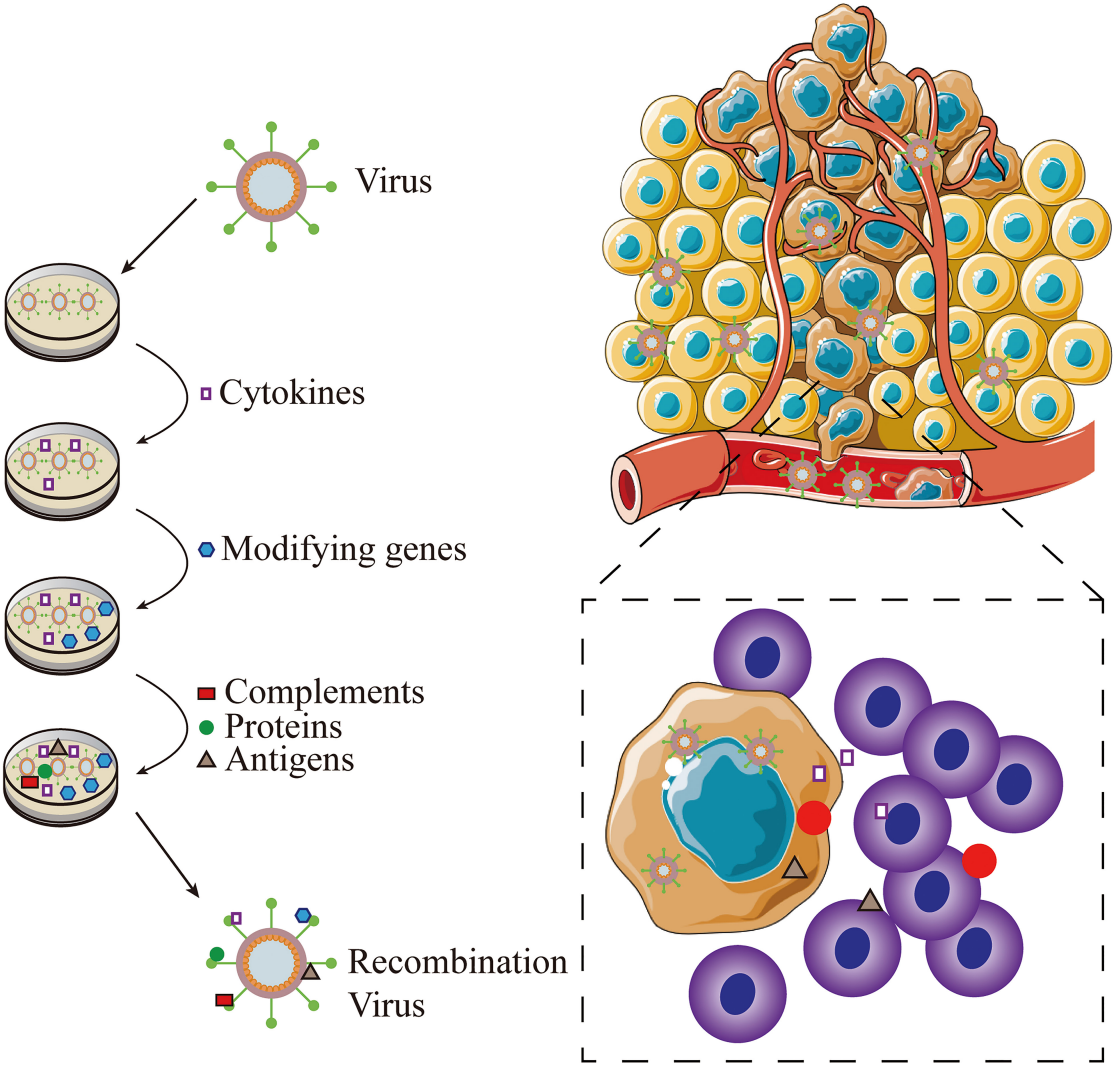
Schematic representation of recombinant OV. OVs can be engineered to achieve different therapeutic purposes by genetic modification, insertion of cytokines, antigen modification, and other means. Engineered OVs often achieve a stronger immune response, recruiting T cells to kill tumors.

Although OVs have produced inspiring results in the treatment of some tumor patients, rationally designing recombinant viruses to express immunostimulatory molecules, cytokines, antigens, and other substances using genetic engineering technology is also expected to improve the effectiveness in the clinics [102–104], as shown in Fig. 5. HSV is easily subjected to rapid recognition and clearance by innate immune effector cells during treatment, which seriously affected the replication and propagation of this virus in tumors [22,105,106]. E-cadherin-expressing target cells are well known to inhibit the cytotoxicity of tumor-activated NK cells by binding to the receptor KLRG1 [107–109], but it is unclear whether virus-activated NK cells could be inhibited. Regarding this, Xu and coworkers [50] engineered an oncolytic HSV to express CDH1, which encoded E-cadherin. The study showed that this recombinant OV suppressed the antiviral cytotoxicity of KLRG1^+^ NK cells by approximately 50%, thus providing sufficient time for OV-CDH1 to complete viral replication, initiate secondary infection, and increase the tumor-killing potency.

It has been extensively accepted that refractory lymphoma treatment using traditional chemotherapy is difficult and unsatisfactory in clinical practice, and immunotherapy is regarded as one of the promising and effective approaches for treating these patients with lymphoma. The combination of cytokine-induced killing cells and DCs with antigenic α-1,3-galactosyl epitope-enhanced lymphoma cell membranes holds high potential. A fascinating treatment outcome was obtained in a clinical study using this design idea [110]. After intravenous administration of the drug, only 6 patients caught fever, but the fever time lasted less than 12 h. This treatment protocol achieved the targeted treatment by integrating the tumor cell membrane with naturally existent antibodies against the α-Gal epitope [111–114]. Among those patients who received the treatment, there were 4 and 3 patients who acquired complete remission and partial remission, respectively. Severe autoimmune disease was not observed in these patients. The number of peripheral immune effector cells was significantly increased at 3 months after treatment, and these results may provide some clues for engineering OVs to achieve homologous targeting and produce recombinant zymogen proteins.

Currently, the combined therapy consisting of α-Gal and OVs has been applied in individual studies [115]. Zhao and coworkers presented an oncolytic heterologous recombinant NDV and elucidated its preparation method and related application. Heterologous recombinant NDV could express α(1, 3) galactosyltransferase [α(1, 3) GT]. Also, they also engineered the recombinant NDV to express fusion genes that carried and manipulated hyperacute rejection antigens, TAAs, and target molecules, which was the first report using an OV as a transduction vector to carry and express the hyperacute rejection antigen gene. This patent established the production technology for a bioconducted hyperacute rejection T cell attack antibody and T cell aggregation antibody, and it solved the key technical bottleneck of anticancer targeting of hyperacute rejection and produced a T cell-directed anticancer effect [116]. This approach laid a solid foundation to OV-based tumor prevention and treatment.

The breakthroughs in OV monotherapy have attracted increasing attentions among many researchers in different communities. To improve the clinical therapeutic effects of OVs, engineered recombinant viruses are the primary research direction in terms of future clinical application of OVs. With the rapid development of genetic engineering technology, OVs offer a variety of treatment options for cancer immunotherapy by modulating their unique biological characteristics.

## OVs in Combination with Other Immunotherapy

OVs have played an irreplaceable role in cancer immunotherapy, and OVs alone have achieved success in the early stage, providing new insights into tumor immunotherapy. With the progressive surge of various immunotherapeutic and oncolytic viral vectors, the combined therapy consisting of OV-based tumor therapy with clinical drug-mediated chemotherapy or immunotherapy has become a research hotspot and acquired remarkable breakthroughs in tumor recession, where OV treatment was highlighted to be the primary contribution [117]. For example, the advent of ICB immunotherapy could serve as the choice to reinforce the clinical antitumor outcome of OVs. Especially, the OV and ICB combined therapy has been validated to be superior to effective than their independent usage in many preclinical studies. This combination approach inhibited regulatory T (T_reg_) cell activation through a nonredundant mechanism and recruited CD8^+^ T cells and NK cells to the TME [8,118].

After administering an OV, PD-L1 expression is up-regulated on the surface of immune cells and tumor cells to evade T cell attack in vivo, which is responsible for tumor resistance and immune escape to OV-based immunotherapy. Wang and coworkers [54] developed an engineered OV as a vector that could coexpress PD-L1 inhibitors and GM-CSF to realize systematic binding and inhibition of PD-L1 on the tumor cell membrane during therapy. Inhibition of PD-L1 by intratumoral injection of this engineered OV promoted their infiltrations and activations of neoantigen-specific T cells and immune cells, and presented neoantigens on tumor cells to effector T cells. The engineered viral vector could release PD-L1 inhibitors to trigger systemic rejections of primary and distant tumors. In brief, this engineered virus expressing the PD-L1 inhibitor improved the efficacy of anti-PD-1/PD-L1-based ICB by activating tumor neoantigen-specific T cell responses.

Intratumoral injection of OVs alone for melanoma treatment has been applied for a long time. Typically, T-VEC as an oncolytic HSV-1 that could encode GM-CSF was approved for the local treatment of advanced melanoma that relapses after initial surgery [17]. According to recent reports, the combined therapy of T-VEC with immune checkpoint inhibitors increases the response rate without affecting the incidence of immune-related adverse events [119]. Based on previous studies, new approaches taking OV as ideal carriers for combined therapy with other systematic drugs have been developed. Very recently, the combination of mitogen-activated protein kinase kinase (MEK) inhibition and T-VEC administration has been explored to kill murine and human melanoma cell lines [120]. Although trametinib, which was chosen as a MEK 1/2 inhibitor, had several side effects (e.g., rash, diarrhea, and fever), this MEK 1/2 inhibitor provided a considerably improved treatment outcome in their study [121,122]. MEK inhibition is known to be related to decreased expression of factors associated with antiviral responses in vivo, e.g., STING (Tmem173) and IFNs. The combined effect of MEK inhibition and OV administration was confirmed to increase the accumulation of CD8^+^ T cells, which further resulted in the production of IFN-γ and granzyme B and an increase in the ratio of CD8^+^/T_reg_ cells in the TME. Although studies performed on xenotransplanted cell lines have certain limitations, these data provide a vital preclinical theoretical basis for the combined therapy consisting of T-VEC administration and MEK inhibition in patients. In a clinical trial for treating advanced melanoma, Chesney and coworkers [123] assessed the value in the treatment of advanced melanoma by using T-VEC in combination with ipilimumab (a novel anti-human cytotoxic T lymphocyte-associated antigen 4 monoclonal antibody).They selected patients with clinical stage IIIB–IV melanoma who had not experienced surgical treatment, where the v-raf murine sarcoma viral oncogene homolog B (BRAF) wild-type (WT) patients receiving no more than one immediate treatment and the BRAF-mutant patients receiving no more than 2 immediate treatments were selected. The eligible patients were then randomly assigned to 2 groups, i.e., combined therapy (T-VEC + ipilimumab) and ipilimumab alone, respectively. In the combined therapy group, T-VEC administration was carried out in the first week (initial dose, ≤4 ml × 10^6^ PFU/ml; after 3 weeks, the dose was adjusted to once per 2 weeks, ≤4 ml × 10^8^ PFU/ml), and after the sixth week, ipilimumab was added (once per 3 weeks, 3 mg/kg) and administered 4 times in total. In the ipilimumab alone (once in 3 weeks, 3 mg/kg) group, ipilimumab was started in the first week and administered 4 times in the monotherapy group. The primary clinical measure was the objective response rate (ORR) that was assessed according to immune-related response criteria. In this cohort of 198 patients with melanoma who received different treatments, the ORR was 39% in the combination arm and 18% in the ipilimumab monotherapy arm [odds ratio (OR), 2.9; 95% confidence interval (CI), 1.5 to 5.5; *P* = 0.002]. This large-scale clinical study provided reliable data for an OV combined with an immunotherapy in patients with solid tumors, and the therapeutic effect was satisfactory beyond expectation.

The new modality of combining OVs with immunotherapy has produced exciting results in both preclinical research and clinical trials. It not only overcomes the limitation of each therapy but also enhances the infiltration of immune cells into the TME. In particular, the activations of neoantigen-specific T cells and immune cells and the presentation of neoantigens on tumor cells were attained. Additionally, this combination with immunotherapy increases IFN-γ and granzyme B expression and the CD8^+^/T_reg_ ratio in the TME, producing unique advantages over OV monotherapy.

Recently, Smac mimetic compounds (SMCs) and OVs were confirmed to synergistically promote tumor recession in cancer-bearing mouse models. SMCs are a group of small molecules designed to antagonize inhibitor of apoptosis (IAP) proteins and sensitize cancer cells to death triggered by inflammatory cytokines, such as tumor necrosis factor α (TNFα). SMC and OV therapies were shown to drive anticancer T cells to respond to them through complementary mechanisms, achieving synergistic treatment of immunogenic tumors [124,125]. Studies in mouse models have demonstrated that SMC therapy indirectly restored the depleted CD8^+^ T cells by targeting TAMs and promoting M1-like polarization. After immunotherapy with SMC/OV, further ICB immunotherapy using anti-PD-1 antibody results in tumor repression in nearly 90% of tumor-bearing mice, demonstrating an excellent therapeutic effect.

## Emerging Technology for Serving OVs in Elevating Antitumor Efficacy and Clinical Translation

### Nanotechnology- or nanomaterial-combined OVs for boosting immunotherapy

**Fig. 6.**
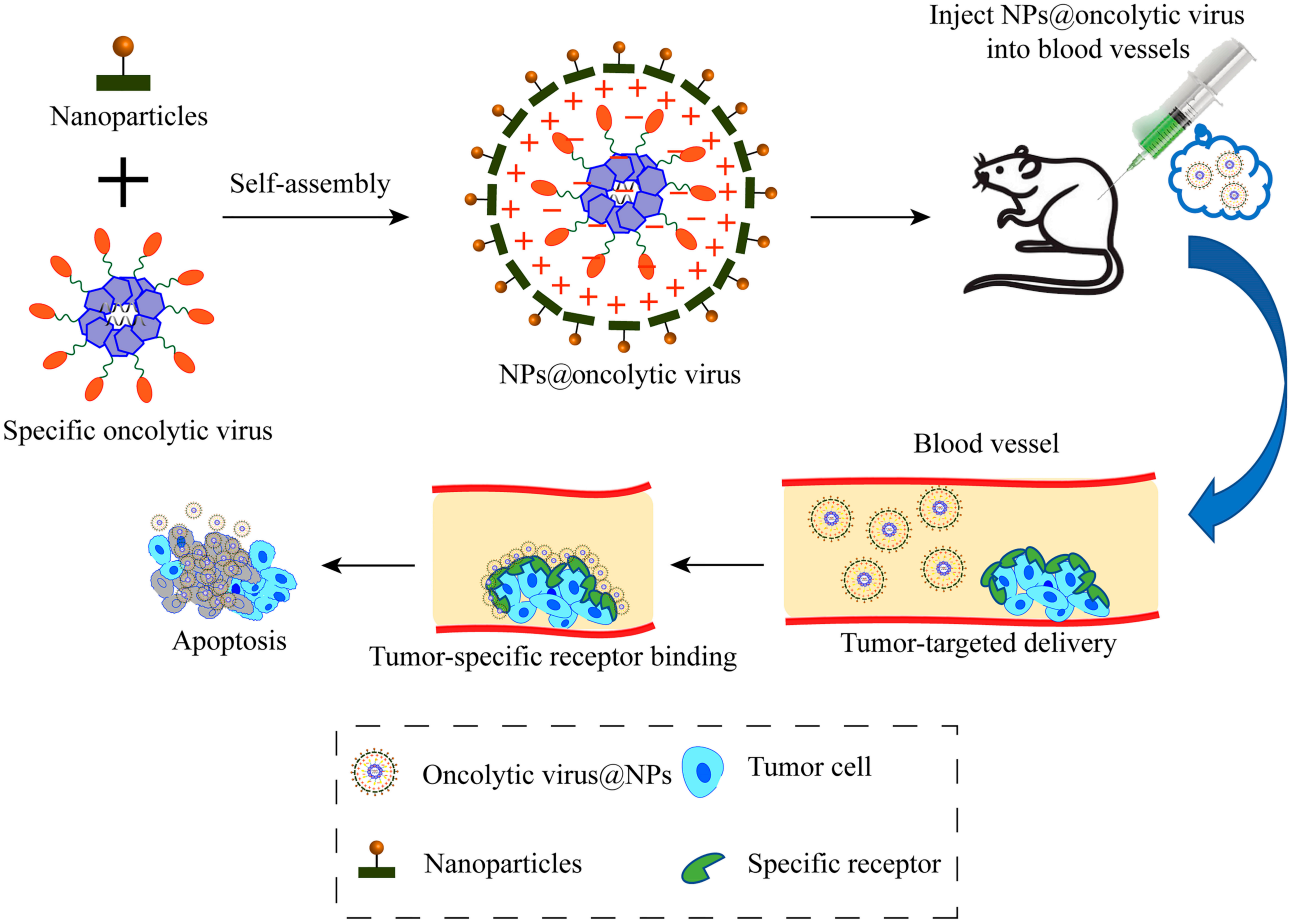
Schematic diagram of oncolysis achieved with an OV combined with a nanoparticle. OVs act as carriers that bind to nanoparticles through self-assembly. After intravenous injection, these complexes target the tumor site, stimulating the body to produce an immune response and exerting a tumor-killing effect.

The current treatment regimens have to address 3 main challenges that malignant tumors are confronted with: progression, recurrence, and metastasis [126–128]. Despite that OVs are on the rise in immunotherapy, nanomaterials are also important research objects that cannot be ignored. Great efforts have been made to develop various nanobiotechnologies and nanoparticles to enhance antitumor effects since these multifunctionalized platforms have versatile purposes, which are available for enhancing OV-based antitumor activity. Generally, nanoparticles refer to photothermal materials [127], magnetocaloric materials [129], and photosensitive materials [7], all of which play significant roles in promoting targeting delivery, elevating antitumor outcomes, and activating systematic immune responses [130–136]. Combining the unique properties of nanomaterials with OV-based immunotherapy will assuredly become a novel therapeutic approach with dual effects, as shown in Fig. 6. This combined strategy is expected to overcome the hurdles of OVs and hold high potentials in clinical translation especially after integration with novel OV engineering, host defense manipulation, and novel target delivery technologies since these factors are essential to achieve the successful cancer therapy.

In a recent study, an oncolytic adenoviral vector (OAD) was encapsulated in an oncolytic nanosphere, which could spontaneously target lung tumor cell line (A549) by the multifunctional principle of polyprotein surface precipitation (PSP) [137]. Ran and colleagues [137] fabricated polyprotein Ad nanospheres with good biosafety and reproducibility through a self-assembly method. The structure of nanospheres was maintained by surface charges and nodules, displaying recognizable quadrilateral or hexagonal morphologies. Based on this PSP technology, the transfection efficiency of viral therapy was ascended; the whole nanospheres could hinder the T cell immune responses to PSP technology during their in vivo transportation process. Concurrently, the extra antigenicity was minimized, and the biological function of the OAD was maintained after release from the nanospheres in vivo [138,139]. In addition, to address the therapeutic limitations of adenoviruses due to the nonspecific targeting and low expression of viral receptors in tumors [140,141], Choi and coworkers [129] designed PEGylated magnetic iron oxide nanoparticles (MIONs) to coat oncolytic adenoviruses. They subsequently used an external magnetic field (EMF) to guide their migration, significantly improving OAD infectivity and specificity. This magnetically responsive complex exhibited excellent oncolytic effects both in vivo and in vitro and overcame the potential safety risk associated with nonspecific liver tropism.

In the decades of research on artificial micro-nano robots, with its good autonomous movement ability, it has broad application prospects in the fields of drug delivery, biosensing, detoxification, and environmental remediation [142]. The Wu team designed a 293T cell-based cellular robot loaded with an OV, modified with targeting peptides and magnetic asymmetric Fe_3_O_4_ nanoparticles on the surface, which could perform magnetically controlled dynamic targeted drug delivery to specifically remove bladder cancer cells. In this robot, 293T cells were used as the virus-loaded carrier, the bladder cancer targeting polypeptide cRGD was fully packaged on the cell surface, and then asymmetric Fe_3_O_4_ nanoparticles were modified to obtain a magnetically driven Janus cell robot (293T-R-Fe@OA). This novel cellular robot can be used as a “Trojan horse” for stealth loading of OVs. As the virus reached the tumor, the virus caused the cell to lyse and then the virus was released into the surrounding cancer tissue to continuously infect other cancer cells, during which virus replication occurs and results in eventual cell lysis. At the same time, this repeated cycle failed to affect normal cells and cause damages to normal tissues, which provided a new treatment idea for the perfusion therapy of bladder cancer [142]. Under the control of an EMF, the cellular robot showed fast and controllable movements in different biological media, and thus, the remote control by magnetic field enabled the cell robot to move directionally in the mouse bladder, which prolonged the residence time of cell motors in bladder cancer. Additionally, the specific targeting of cRGD further enhanced the accumulation of cellular motors in bladder cancer. The 2 enhanced affinity strategies greatly improved the tissue penetration and anticancer capabilities of the Janus cell robot in 3D bladder tumor cell spheroids and orthotopic mouse bladder tumor models. It provided a clever and typical design idea for the combined application of OVs and nanomaterials, and also contributed to the clinical application of micro-nano robots in the future.

Collectively, the marriage application of OVs in combination with nanoparticles or nanobiotechnologies is still under way but remain at their infancy. However, materials featuring different physical and chemical properties will absolutely bring new effects and inspirations to traditional single agent-containing OVs. At present, with the rapid development of these 2 modalities, we believe that the combination of these 2 technologies will make important advances. Moreover, there is no doubt that more applications for adenoviral vectors are expected [24], because researchers can use the rich biological properties of oncolytic adenoviruses. Notably, the application of other viral vectors in combination with nanoparticles may be another direction for researchers to explore. Given that current most delivery methods are passive, exploring an active delivery method may improve OV efficiency and facilitate the targeting and dissemination of engineered OVs.

### CRISPR-based reliable primate model bearing primary liver cancer for driving clinical translation

With the rapid progress of clinical research, selecting more advanced experimental animals whose genetic structure is similar to that of humans is an important and reliable research model, which can assist researchers to explore the pathogenesis of diseases and evaluate the feasibility of preventative and therapeutic methods [143]. Nonhuman primates (NHPs) are the most reliable animals for the development of new drugs used in clinics [144]. Cynomolgus monkeys are one of the NHPs with advanced evolution level, and their function, structure, and response approximately approach those of humans, making them an ideal preclinical experimental animal model [145–150]. Unfortunately, the absence of spontaneous orthotopic tumor model hinders this progression of new drugs. Zhong and coworkers [151] evaluated ultrasound-guided infusion of CRISPR-Cas9 into the liver of cynomolgus monkeys via the intrahepatic portal vein. They demonstrated that Pten and p53 gene mutations could be efficiently generated in the liver of these monkeys through intrahepatic portal vein injection of the CRISPR-Cas9 genome editing system. This new method overcomes the risks of traditional methods used to generate animal models with genetic mutations and bypasses the demands of breeding multiple mutant animals, providing a somatically mutated NHP model of human primary and metastatic liver cancer for translational study. The research data obtained on this NHP model provide new models and clues for gene therapy mediated by OVs and other therapeutic methods, convincingly indicating the potential of this preclinical treatment. In addition, this technology can be used not only to establish liver disease models such as fatty liver, cirrhosis, and hepatitis but also to deliver the CRISPR-Cas9 system to other organs (such as lung and kidney) under ultrasound guidance through relevant blood vessels to establish corresponding disease models for the study of new gene diagnosis and drug discovery and other therapeutic strategies. According to the researchers, the cynomolgus monkey facility was notarized by the Association for Assessment and Accreditation of Laboratory Animal Care (AAALAC) International and all experimental procedures.

## Clinical Application of Some Typical OVs

**Fig. 7.**
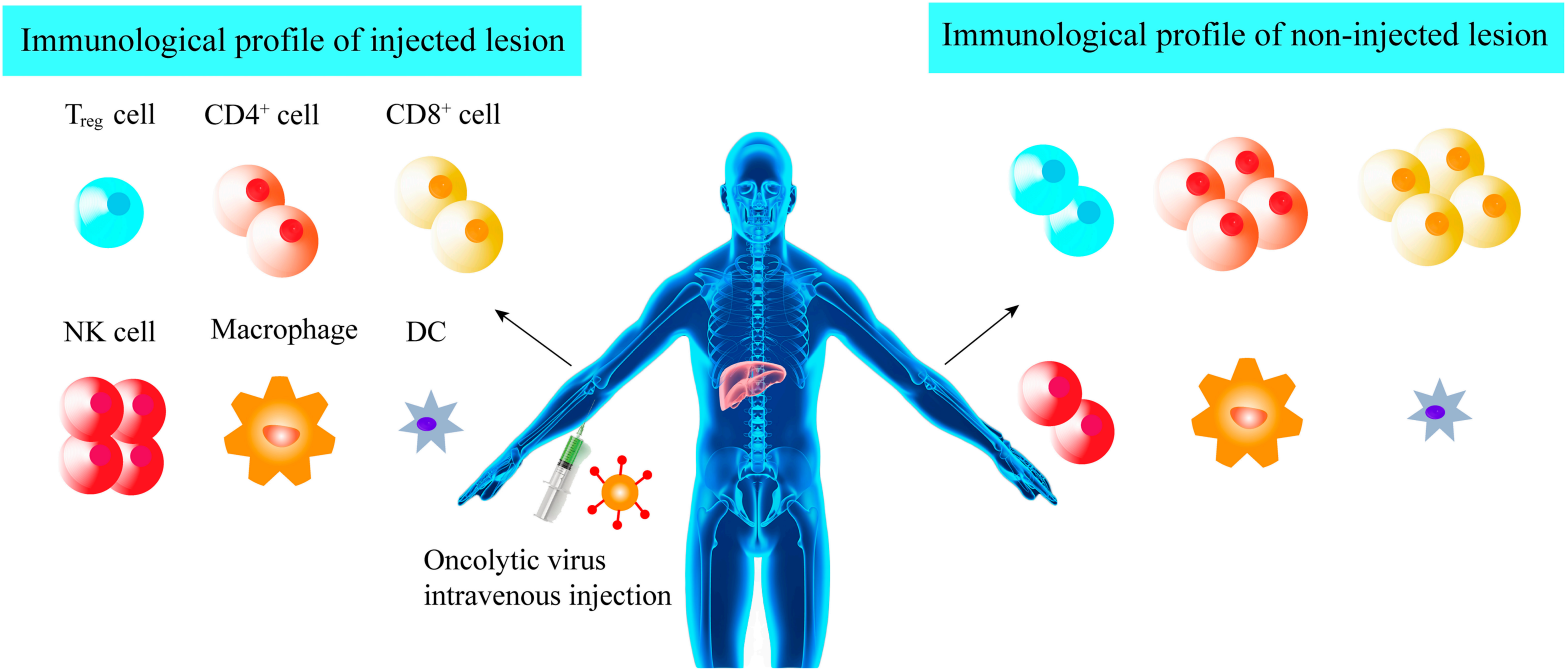
Multimodal mechanisms of action associated with OV therapy. OVs can be injected into accessible lesions and mediate tumor rejection in both injected and distant, non-injected lesions. The immune response in injected lesions is dominated by an influx of NK and CD8^+^ T cells with a reduction in T_reg_ cells. In the non-injected left lower extremity tumor, no virus is present, and the immune response is dominated by CD4^+^ T and NK cells. Macrophages and dendritic cells (DCs) are also present, although these cells have not yet been adequately characterized in this setting. Other factors might also influence the activity of OVs.

Exploration and evaluation of a variety of OVs are ongoing in clinics (Table 2) despite that most OVs are still in the early clinical trials. In the clinics, no matter what administration method, e.g., intravenous or local injection, was used, these immunotherapeutic agents can ignite the body to generate robust immune responses and achieve convincing and desirable oncolytic effects, as shown in Fig. 7 [152]. Here, we discussed the developments and applications of key OVs in recent clinical trials and presented some typical representatives of clinical or preclinical studies.

**Table 2.** Progress of oncolytic viruses in clinical trials.

**Virus type**	**Virus name**	**Vaccine characteristics**	**Cancer type**	**Injection pathway**	**Combined type**	**Phase, study type**	**Status**	**Trial number**	**Ref.**
Adenovirus (DNA virus)	CG0070	Specific promoter; GM-CSF	Bladder cancer	Intravesical	Immune checkpoint modulation	Phase 2	Active, not recruiting	NCT02365818	191
	DNX-2401	Δ24-RGD insertion	Glioblastoma, glioma, astrocytoma, gliosarcoma	Artery	Surgery	Phase 1/2, open label, nonrandomized	Active, not recruiting	NCT03178032	52
	VCN-01	Dose escalation of VCN-01	Retinocytoma (recurrent)	Intratumoral	None	Phase 1, open label, single group	Recruiting	NCT03284268	192
	Onyx-015	Type 2/5 chimera, E1B deletion	Lip cancer and oral cancer, head and neck cancer, oropharyngeal cancer	Intratumoral	Cisplatin, fluorouracil	Phase 1	Withdrawn	NCT00006106	193
	H101	E1B deletion, partial E3 deletion	Refractory malignant ascites	Intraperitoneal	Chemotherapy	Phase 2, open label, single group	Recruiting	NCT04771676	162
	Colo-Ad1	PsiOxus therapeutics	Colon cancer, non-small cell lung cancer, bladder cancer, renal cell carcinoma	Intratumoral and intravenous	None	Phase 1, open label, single group	Completed	NCT02053220	194
	ProstAtak	TK insertion	Prostate cancer	Intraprostatic	Valacyclovir	Phase 2, randomized, parallel	Active, not recruiting	NCT02768363	195
	Oncos-102	Δ24-RGD-GM-CSF insertion	Prostate cancer	Intratumoral	DCVAC/PCa \(phase 1), cyclophosphamide (phase 2)	Phase 1/2, open label, single group	Terminated	NCT03514836	196
	LOAd703	Encoding TMZ-CD40L and 4-1BBL	Pancreatic cancer	Intratumoral	Nab-paclitaxel	Phase 2, open label, nonrandomized	Recruiting	NCT02705196	197
	NG-641	Expresses fibroblast activation protein (FAP)	Metastatic epithelial tumor	Intravenous	None	Phase 1, open label, single group	Recruiting	NCT04053283	198
	NG-350A	Expresses agonistic CD40 antibody	Metastatic epithelial tumor	Intravenous	None	Phase 1, single group	Recruiting	NCT03852511	199
Vaccinia virus (DNA virus)	PexaVec (JX594)	TK deletion, GM-CSF insertion	Metastatic cancers and advanced cancers	Intratumoral	Ipilimumab	Phase 1, open label, single group	Recruiting	NCT02977156	200
	GL-ONC1	Deletions in F14.5 L, thymidine kinase and A56R	Ovarian cancer, peritoneal carcinomatosis, fallopian tube cancer	Intraperitoneal	Chemotherapy and bevacizumab	Phase 1/2, open label, single group	Active, not recruiting	NCT02759588	201
	ASP9801	Encoding IL-7 and IL-12	Metastatic cancer, solid tumors, advanced cancer	Intratumoral	Pembrolizumab	Phase 1, open label, nonrandomized	Recruiting	NCT03954067	202
	RGV004	Encoding CD3/CD19 bispecific antibody	B cell lymphoma	Intratumoral	None	Phase 1, open label, single group	Active, not yet recruiting	NCT04887025	203
Herpesvirus (DNA virus)	OrienX010	ICP34.5 deletion, ICP47 deletion, GM-CSF insertion	Melanoma	Intratumoral	JS001	Phase 1, open label, single group	Recruiting	NCT04206358	204
	HF10	UL56 deletion, single copy of UL52	Melanoma	Intratumoral	Nivolumab	Phase 2, open label, single group	Terminated	NCT03259425	205
	G207	ICP34.5 deletion, UL39 disruption	Glioblastoma, glioma, astrocytoma	Intratumoral	None	Phase 2, open label, single group	Not yet recruiting	NCT04482933	206
	T-VEC	ICP34.5 and ICP47 deletion, human GM-CSF insertion	Melanoma	Intratumoral	None	Phase 2, open label, single group	Recruiting	NCT04427306	207
	SEPREHVIR(HSV1716)	ICP34.5 deletion	High-grade glioma	Intratumoral	None	Phase 1, open label, single group	Terminated	NCT02031965	208
	OH2	Insert the human GM-CSF gene	Pancreatic cancer	Intratumoral	None	Phase 2, open label, single group	Recruiting	NCT04637698	209
Reovirus (RNA virus)	Reolysin	Transgenic expression of yeast cytosine deaminase (Toca511)	Breast cancer	Intravenous	Retifanlimab	Phase 2, open label, single group	Recruiting	NCT04445844	210
Measles virus (DNA virus)	MV-NIS	Transgenic expression of carcinoembryonic antigen and thyroidal NIS	Myeloma, endometrial neoplasms	Intraperitoneal	F-18 TFB	Phase 1, open label, nonrandomized	Completed	NCT03456908	211
	MV-s-NAP	Expresses *Helicobacter pylori* neutrophil activating protein	Breast carcinoma	Intratumoral	None	Phase 1, open label, single group	Recruiting	NCT04521764	212
Coxsackie viruses (DNA virus)	Cavatak (CVA21)	Wild-type coxsackie viruses A21	Non-muscle-invasive bladder cancer	Intravesical	Mitomycin C	Phase 1, open label, nonrandomized	Completed	NCT02316171	213

### Herpes simplex virus 1

Glioblastoma (GBM) is a highly refractory malignant tumor. According to the 5-year survival rate data of the National Brain Tumor Society of the United States, the 5-year survival rate of GBM patients in the United States from 2009 to 2015 is only 7%, which is lower than the “king of cancer” pancreatic cancer (PCA), which is the brain malignancy with the lowest survival rate [153]. The traditional GBM treatment can prolong the median overall survival (OS) of patients to 20 months through postoperative adjuvant chemotherapy and radiotherapy with temozolomide, and the emerging tumor electric field therapy (TTF) that has emerged in recent years [154]. However, patients still have a very high chance of recurrence after treatment. At this time, Todo and colleagues [155] started an OV antitumor study against GBM and achieved exciting results. In this clinical study, the OV G47Δ used by Japanese researchers is a third-generation HSV prepared by genetic engineering. Its ability to kill cancer cells is further enhanced, and the treatment safety is good. In phase 1 clinical studies, some GBM patients have a survival period of nearly 4 years. The median OS of patients receiving continuous injections of HSV G47Δ reached 28.8 months, which far exceeded the data of previous treatments [156]. It is cumbersome to use G47Δ during the treatment process, and it is necessary to use stereotaxic minimally invasive surgery to inject the virus into the tumor site of the patient, and complete 6 injections within 5 months. Based on considerations of medical ethics and related regulations, the researchers did not set up a control group and only used a single-arm study to evaluate the efficacy. At the beginning of the treatment phase, the researchers did not set high goals in the face of such intractable GBM, and they would consider a 1-year survival rate of 40% for the treated patients to be an ideal result, because the GBM that relapses after chemotherapy can only achieve a 1-year survival rate of 15% with previous treatment. But the final result was striking. As of March 2022, the 1-year survival rate of 19 patients who received treatment was 84.2%, and the median OS after starting G47Δ treatment was only 20.2 months, which is far beyond the history. If calculated from the time of initial surgery, the median OS was as long as 28.8 months. At the same time, the treatment safety of G47Δ is also very good. The main side effects are fever, nausea, vomiting, and lymphopenia, but the incidence of treatment-related adverse events of grade ≥3 is only 26.3%, and it can be relieved by itself without intervention. Only one patient required longer hospitalization due to more severe fever.

Compared with previous treatment regimens, whether it is targeted therapy such as temozolomide chemotherapy/radiochemotherapy, bevacizumab, or PD-1/L1 inhibitor therapy, the median OS for recurrent GBM is generally only 5 to 10 months, which is far from the effect of G47Δ treatment used in this study, and 5 patients have survived for more than 3 years after G47Δ treatment, which is extremely difficult to achieve with traditional treatment. All in all, G47Δ has performed very well in the treatment of recurrent GBM, and it was officially approved for marketing in Japan last June. However, after all, this is a small-sample phase 2 clinical study. If such excellent performance can be successfully reproduced in the phase 3 study, or the combination of immunotherapy can be successful, it will be a great boon for GBM patients.

T-VEC, a genetically modified HSV-1 strain, was designed to selectively replicate in tumors and produce GM-CSF, which enhanced the release and presentation of antigens and activated the systemic antitumor immune responses [157]. In a phase 3 clinical study, compared with subcutaneous injection of GM-CSF alone, intratumoral injection of engineered T-VEC into melanoma metastases considerably improved the survival time of patients bearing advanced melanoma [17]. Ribas and coworkers [119] tested the effects of T-VEC therapy on cytotoxicity, T cell infiltration, and therapeutic efficacy of the anti-PD-1 antibody (i.e., pembrolizumab) in a phase 3 study of T-VEC combined with pembrolizumab. In 21 patients receiving this treatment, the levels of several cell subsets in their tumors were changed, e.g., the increases of CD8^+^ T cells, PD-L1 protein expression, and IFN-γ gene expression (ClinicalTrials.gov: NCT02263508). These observations show that OV therapy can enhance the efficacy of anti-PD-1 therapy by changing TME. Unfortunately, this study of T-VEC plus anti-PD-1 failed in phase 3. Despite this, the posttreatment clinical data can provide instructive insights into future combined research based on T-VEC.

The improved prognosis of melanoma patients treated with T-VEC sufficiently demonstrates the vital role of OVs in anticancer therapy. However, the development of OVs that function as therapeutic agents deserves more attentions. In particular, appropriate clinical trial design and dosing regimen determination, systematic pharmacodynamic analyses, research and development program establishment to address biosafety concerns, and new manufacturing and regulatory processes should be focused on. Likewise, meticulous consideration of choosing individual patients is also essential. In detail, immunocompromised patients are not be the best candidates because OV-mediated antitumor immunity may be compromised in these patients.

### Adenovirus

Adenovirus is a medium-sized virus with an icosahedral protein capsid that contains 36,000 base pairs of linear double-stranded DNA. After adenoviruses entered host cells, they were quickly replicated and assembled to produce 10,000 progeny viral particles [158]. In the treatment of solid tumors, oncolytic adenoviruses show high beneficial application potential [159]. The clinical translation and development of oncolytic adenoviruses are being strongly pursued by researchers.

As the first commercialized OV product worldwide, Oncorine is mainly used for head and neck cancer and, at the same time, extended to some indications in liver cancer, lung cancer, PCA, and malignant pleural effusion. For hepatocellular carcinoma (HCC), less than 20% of HCC patients are eligible for surgical resection or liver transplantation. Transhepatic artery chemoembolization (TACE) is currently recognized as the best treatment option worldwide [160]. Lin and coworkers [161] used recombinant human type 5 adenovirus H101 in combination with chemotherapy in a clinical study for HCC treatment. Therein, patients with unresectable liver cancers were divided into several groups and accordingly received arterial injections of Oncorine and TACE (Oncorine group, *n* = 87) or TACE alone (control group, *n* = 88) as their treatment protocols. During this study, Child–Pugh class A and B patients did not experience any serious complications after H101 treatment but did experience normal liver function loss. Moreover, the difference in liver toxicity between the treatment groups only existed for 1 to 2 months and displayed neglectable significance. However, high fever and increased white blood cell counts were frequently observed in the patients in the H101 treatment group, which were probably caused by the virus-induced immune activation in the body. Additionally, fever arose from the production of inflammatory cytokines after oncolytic adenovirus infusion via the hepatic artery [162]. In this controlled study, results indicated that the antitumor effect of H101 was obvious, and clinical follow-up visit studies preliminarily revealed that drugs could improve the survival time of patients. Despite acquiring inspiring results, randomized controlled and long-term studies remain urgently desirable to prove the actual effects of these drugs in clinics.

### Reovirus

Reovirus is a virus with a segmented double-stranded RNA genome, which is widely distributed. The virion is spherical in shape and has an octahedral structure with a size between 60 and 80 nm. Its total genome size is 23.5 kilo–base pairs, and the genome can be divided into 10 fragments [163,164]. The overexpression of oncogene Ras and the impaired type I IFN signaling endow reovirus with robust activity in cancer treatment [165]. These extraordinary characteristics determines that reovirus is regarded as one of the most interesting viruses.

Noonan and coworkers [166] conducted a phase 2 clinical study (NCT01280058) for evaluating the pretreatment outcome of metastatic PCA after receiving the OV pelareorep (Reolysin) treatment. In this study, patients were randomly assigned to several groups that received pelareorep, carboplatin, and paclitaxel (group A) or carboplatin and paclitaxel (group B) at a ratio of 1:1. During the treatment cycle, progression-free survival (PFS) and the RECIST 1.1 standard ORR were monitored, and authors found that introducing pelareorep to carboplatin and paclitaxel failed to improve PFS. The immune status of patients was assessed before the third treatment cycle, and the neutralizing anti-reovirus antibody titer was assessed in group A [167,168]. The final data showed that many immune biomarkers related to disease control rate (DCR) or PFS were detected in both treatment groups, e.g., the proliferations of NK cells or B cells and KRAS mutations. Pelareorep appeared to be safe and well tolerated. Except for the occurrence of reversible lupus nephritis in the pelareorep group, there were no significant differences between the 2 groups in terms of toxicity and quality adjustment time without disease symptoms. Although the desired therapeutic outcome was not reached, pelareorep was found to induce cytopathic effects in those cells carrying KRAS mutations, which was meaningful and deserved to be highlighted since 70% to 90% of PCA cases have KRAS mutations through predecessors [169,170]. Based on this point, conducting highly feasible clinical trials are valuable. Notably, the inconsistency or difference away from clinical expectation was probably attributed to neutralization of pelareorep by other unknown mutations, leading to the failure of verifying its real efficacy in clinical trials.

### Newcastle disease virus

The immunogenicity induced by NDV could be recognized at an early stage, and many studies have explored this virus to induce immunity in patients through the administration of virus-modified cancer cell vaccines [171–174]. Many early studies were conducted by Cassel and colleagues, and they adopted autologous or allogeneic neocoronavirus tumors to vaccinate patients with high-risk melanoma who underwent resection. Results indicated that the OS rate was improved in the vaccinated patients compared with historical controls [175–177]. Some studies have confirmed that NDV infected cancer cells, enhanced the immunogenicity of cancer cells, and had the potential ability to stimulate antitumor immunity.

Due to the cumbersome preparation procedures that autologous virus-modified vaccines required, many studies have explored the direct usage of NDV in cancer patients. In the first report using NDV in humans, the leukemia cell count was decreased in a patient with acute myeloid leukemia who received the treatment with the Hickman strain of NDV [178], and his clinical symptoms were improved temporarily. Additionally, Csatary and colleagues [179] also reported a series of treatment cases in which the NDV strain (MTH-68) was directly used to treat various advanced cancer-bearing patients via different administration routes. Inspiringly, partial or complete recession was observed in some cancers. In another series of studies, 14 patients with GBM received intravenous injections of the NDV strain MTH-68 at different times. Seven of the patients responded to this treatment, and 4 of them survived for 5 to 9 years at the time of publication in 2004 [179]. The intravenous administration of a recombinant NDV strain expressing GM-CSF (MEDI5395) based on the 73-T strain in combination with durvalumab (NCT03889275) is being evaluated in patients with various advanced malignancies. More noninfectious viruses after genetic engineering are situated in different development stages and are expected to enter the clinic within the next year. At present, few human recombinant NDV has entered clinical applications yet, which can be attribute to the intractable virus gene engineering [179]. As the genome editing technology such as CRISPR-Cas technology advances, NDV with more functions in clinics is expected in the near future.

### Coxsackie viruses

OVs have incomparable advantages over other therapies for tumor treatment. In recent years, besides the widely studied oncolytic adenoviruses and HSVs, coxsackie virus has become a new promising candidate virus with particularly valuable characteristics [180]. Coxsackie virus B3 (CVB3) was first reported in 1957 [181], but it was not taken seriously by researchers because they did not find certain characteristics. Recently, Miyamoto and colleagues [182] described the powerful antitumor efficacy of CVB3 in mice with lung cancer. Furthermore, some studies have demonstrated the antitumor efficiency and biological safety of CVB3 and elucidated the potential antitumor mechanisms in in vitro and preclinical experiments.

Coxsackie viruses known as a single-stranded small RNA enterovirus has shown impressive antitumor efficacy in lung cancer, breast cancer, and melanoma in preclinical studies [183]. Based on previous clinical safety studies, Andtbacka and colleagues [184] used coxsackie viruses A21 (V937) to conduct the Managing Cancer And Living Meaningful (CALM) trial in patients with unresectable stage IIIC or IV melanoma, and they evaluate the effects of V937 on the immune heterogeneity of host tumors and surveyed the antitumor efficacy (NCT01636882). In this phase 2 clinical study, the authors classified solid tumors according to the immune-related response assessment criteria (irRECIST). A total of 38.6% of patients whose complete relief (CR), partial relief (PR), and stable disease (SD) were recorded survived for 6 months or longer, and the median overall PFS time according to Kaplan–Meier analysis (including patients who withdrew from the review 6 months ago) was 5.7 months. The results showed that intratumoral V937 was well tolerated, and also suggested that V937 had a systemic antitumor activity and merited further investigation. One survival result exceeded the Korn benchmark specified in the agreement [185]. Further studies using V937 for other cancers and immune checkpoint inhibitors for melanoma are ongoing. Most oncolytic coxsackie viruses are tested to be sufficiently tolerated in the body, but occasional side effects still occur. To avoid side effects, genetic engineering should be adopted to make these viruses sensitive to tissue-specific or tumor suppressor miRs.

### Vaccinia virus

As a double-stranded DNA virus, vaccinia virus (VV) is a member of the *Orthopoxvirus* genus in the Poxviridae family, and it has unique biological characteristics. Differing from other DNA viruses, VV always exists in the cytoplasm in the entire infection cycle from the virus infection or entry into cell to progeny virus replication [186]. Depending on this, the virus’s DNA is not integrated into the host genome [187]. As a result, VV will never induce tumor and instead will cure tumor, features of good biological safety. Typically, Nakao and coworkers [56] proposed an oncolytic VV platform to simultaneously express IL-7 and IL-12 to change the immunosuppressive status in solid tumors. This genetically engineered oncolytic VV not only could serve as a monotherapy but also can unite with ICB-based targeting immunotherapy. Local injection of hIL-7/mIL-12-VV increased the infiltration of immune cells, enhanced the expression of major histocompatibility complex class II on APCs, and up-regulated the activity of multiple immune-related pathways, consequently inducing antitumor immune activation in noninjected distant tumors. Concurrently, the viral genome was not detected by quantitative polymerase chain reaction (qPCR). However, the authors did not confirm whether tumor-specific memory T cells infiltrated into the rechallenged CT26 tumors, but WT tumor cells were completely rejected in this experiment. Since the antitumor memory reactivation and the inflammation level in the TME represented by the presence of infiltrating lymphocytes closely correlated to tumor prognosis and affected the OS rate and PFS, reactivating the memory effect and attenuating inflammation level are highly desirable development directions in designing OVs [188–190].

## Perspective, Challenge, and Outlook

Since OVs have been proven to be a powerful immunotherapy approach in recent clinical trials, they have been identified as promising novel agents for tumor therapy. Nevertheless, it is confessed that immune escape and immunologic surveillance failure of tumor cells remain the major challenges in OV therapy. Almost all aspects of the human immune indexes are affected by OVs, and thereby, the antitumor activity of these agents could enhance tumor immunotherapy through various mechanisms. Inspired by the current cutting-edge progress and antitumor outcomes in preclinical or clinical trials, researchers and doctors believe that OVs are one of the novel, ideal, and potent immunotherapeutic agents for clinical tumor treatment. However, the specific action mechanisms of OVs in vivo remain incompletely understood and need more investigations and advanced tools to systematically understand their action mechanisms that not only include immune activation. The adequate understanding of action mechanisms is favorable for developing or engineering specific OVs objective to some tumor with different overexpressed targets.

In genetically engineered OVs, the replication and expansion or propagation of OVs are pivotal, which dictate the fate and treatment outcome of OVs. Due to the failure of continuous replication and expansion of OVs, one injection fails to acquire satisfied oncolytic consequence, which needed multiple injections or combined therapy with other therapeutic methods. However, there is still a doubt on whether the multiple injections will pose tumor resistance, akin to chemotherapeutic drugs. This direction has not been explored yet, which, however, is highly necessary to be investigated. Additionally, there are many significant species differences between mice and human. Although systematic and deep signaling pathways and treatment mechanisms are understood on the mice model, they maybe not appropriate for assessing human. Therefore, reassessment on primate model that physiologically approaches human is preferable, which not only serves for drug development but also acts as the reliable model to assess the functions and mechanisms of OVs and accelerate the druggability of OVs in clinical translation.

Although OVs are available for use in combination with various other therapies, ICB immunotherapy is still recognized the primary choice in early clinical trials to produce an increased therapeutic response without increased toxicity by taking into consideration immunotherapy. Therefore, there is a long way to drive more OVs as immunotherapeutic agents to enter clinical trials. We have reviewed partially completed or incomplete OV clinical trials, and it is not difficult to find that any single therapy has been difficult to meet the current clinical treatment needs. Most trials used OV in combination with other modalities to treat tumors. Inspired by the combined therapy and the significant advances of nanomedicine and nanotechnology, the combination of OVs with nanomedicine or nanotechnology will be the candidate direction of improving OV-based antitumor efficacy. Based on nanotechnology and nanomedicine, various treatment methods (e.g., sonodynamic therapy, photodynamic therapy, and photothermal ablation) have been established and combined with chemotherapy, interventional therapy, and immunotherapy, which received remarkable outcomes. Very recently, there are several cases that combined nanomaterials with OVs, such as acoustic, chemical, photodynamic, and oncolytic bacteria. These combined applications significantly improved the delivery efficiency of OV, further enhanced the immune response, and showed a good therapeutic effect. In light of the fact that nanomaterials or nanotechnology has been demonstrated to activate systematic immune responses and favor immunotherapy, this combined strategy of OVs with nanomaterials as a promising direction will arouse more and more attention.

It is believed that the activated immune responses and other antitumor actions such as thrombus and direct killing ability propel OVs or recombinant OVs to become one of the first-line antitumor methods sooner or later when the dose, administration route, and treatment duration of OVs were optimized and figured out. Further verification on the neoantigen-specific T cell pool activated by OVs is also an essential research objective. With the rapid development of OVs, various genetically modified or engineered agents have emerged in humans. OVs with favorable biosafety and potentiated immune effects are widely accepted as ideal agents for optimizing OV-centered combination immunotherapy. It is expected that OVs will provide more exciting clinical results as well as a new standard therapy for cancer patients in future studies.
